# Editorial: If maternal breastfeeding is not possible: exploring safe and sustainable infant feeding options in all contexts

**DOI:** 10.3389/fnut.2025.1657289

**Published:** 2025-07-22

**Authors:** Alessandro Iellamo, Bindi Borg, Julie P. Smith, Seema Mihrshahi

**Affiliations:** ^1^FHI 360, Washington, DC, United States; ^2^Department of Health Systems and Populations, Macquarie University, Sydney, NSW, Australia; ^3^Health Research Institute, University of Canberra, Canberra, ACT, Australia; ^4^Crawford School of Public Policy, The Australian National University, Canberra, ACT, Australia; ^5^School of Public Health, University of Sydney, Sydney, NSW, Australia

**Keywords:** infant feeding in emergencies, IYCF-E, IYCF, wet nursing, donor milk, breast milk expression, breastfeeding counseling, maternal breastfeeding

## Introduction

Breastfeeding is the biological and normative standard for infant and young child feeding. It has been proven to reduce infant mortality and morbidity, enhance immunity, and support cognitive development (1–5). However, in both development and humanitarian contexts, there are circumstances in which maternal breastfeeding is not possible due to maternal death, illness, separation, or cultural and structural barriers (6, 7). The infant separated from their mother who is incarcerated or in detention should also be considered (8, 9). In such situations, the critical question is how to ensure that infants receive safe, appropriate, and sustainable alternatives to maternal breastfeeding (7).

This *Frontiers in Nutrition* Research Topic, titled “*If maternal breastfeeding is not possible: exploring safe and sustainable infant feeding options in all contexts,”* brings together a set of pioneering contributions that examine ethical, operational, and scientific considerations in non-breastfed infant feeding. The studies emphasize that every infant has the right to safe nutrition, and that human milk whether maternal, expressed, donated, or wet-nursed—must remain the preferred option (Abdelrahmman et al., Conboy-Stephenson et al., Pramono and Hikmawati, Gawrońska et al.).

## The evidence: breastfeeding and breastmilk over commercial milk formula

Evidence overwhelmingly supports the superiority of breastfeeding over breastmilk substitutes (BMS), in terms of survival and health outcomes in both high and low-resource settings Gawrońska et al., (10–31). This also applies to the preferability of breastmilk or donor milk feeding. A comparative analysis of feeding options confirms that alternatives such as wet nursing, maternal expressed breastmilk, and donor human milk (DHM) provide far greater protection against morbidity and mortality than formula. Infants who are not breastfed face increased risks of diarrhea, pneumonia, sudden infant death syndrome (SIDS), necrotizing enterocolitis, and long-term conditions such as obesity and type 2 diabetes in all country settings Gawrońska et al., (10–31). Wet nursing, particularly when culturally accepted and properly screened, offers raw, immunologically active milk. Because it is a closed biosystem of mouth-to-nipple, it eliminates the risks associated with expressing, storing, transporting and feeding expressed breastmilk. For example, WHO guidance, including during the COVID-19 emergency is that in appropriate circumstances, a suitable wetnurse was an option if the mother was too ill to breastfeed (32) or the new UNICEF Technical and Operational Guidance on Supporting Wet Nursing (33), provides practical suggestions on how to support wet nursing practices in a humanitarian context. There is a need for more systematic country level guidance on implementing this (34). Similarly, maternal expressed milk remains highly protective and practical where direct breastfeeding is interrupted, though the process of expressing and feeding breastmilk introduces potential contamination risk. DHM when correctly screened, pasteurized when used through milk bank, needed, delivered and fed in a cup offers substantial protective value, especially in neonatal and emergency settings Gawrońska et al., (10–31). In contrast, BMS poses significant risks, especially in emergencies, including dependency on supply chains, unsafe preparation, and loss of critical immunological protection (35–39).

## OG-IFE: global guidance in emergencies

The *Operational Guidance on Infant and Young Child Feeding in Emergencies* (OG-IFE), developed by the Emergency Nutrition Network (ENN) and the Infant Feeding in Emergencies Core Group (IFE CG) (7) provides the World Health Assembly endorsed framework for safe infant feeding during crises (40, 41). OG-IFE underscores that breastfeeding should be protected, promoted, and supported at all levels of emergency preparedness and response. When breastfeeding is not possible, the guidance prioritizes the use of alternatives in the following order: re-lactation, wet nursing, maternal expressed breastmilk, donor human milk, and lastly, infant formula only under strict criteria with full support and monitoring. Humanitarian agencies should not risk being the Trojan horse for industry marketing in emergencies.

OG-IFE also emphasizes the critical importance of appropriate complementary feeding from 6 to 23 months and the regulation of BMS, including prohibition of donations and adherence to the International Code of Marketing of Breast-Milk Substitutes (42). Multisectoral coordination, particularly with health, WASH, protection, and food systems is essential to enable safe infant feeding responses (7). In particular, experience shows that mother-baby areas are critical for protecting, promoting and supporting IYCF practices in emergencies (43–45).

## Highlights of this Research Topic

This Research Topic features four contributions that significantly advance the understanding and operationalization of infant feeding options beyond maternal breastfeeding.

### Wet nursing: culture, risk, and implementation needs

Abdelrahmman et al. explore the acceptability and implementation barriers of wet nursing in emergencies. Drawing on interviews with breastfeeding counselors, the authors highlighted seven themes including HIV-related stigma, trust issues, lack of protocols, and cultural perceptions of wet nursing. The study calls for the integration of wet nursing into emergency protocols with safeguards, informed consent, and culturally sensitive counseling.

### Preserving donor milk quality

Conboy-Stephenson et al., in their review, examine how different processing technologies affect the microbiological safety and nutritional integrity of donor human milk. Holder pasteurization, though widely used, significantly reduces lactoferrin, IgA, and other immune components. Importantly for low resource settings and emergencies, the authors advocate for low-impact technologies that preserve more of the milk's bioactivity without compromising safety.

### Media and public perception in Indonesia

Pramono and Hikmawati conduct a media content analysis of donor human milk discourse in Indonesia. Their findings reveal that while some narratives support donor milk, widespread misinformation and limited regulation generate public confusion and resistance. The authors recommend improved communication strategies tailored to religious and cultural contexts.

### Resilient milk banking in emergencies

Gawrońska et al. compare Poland's milk banking response to COVID-19 and the Ukrainian refugee crisis. The study shows how decentralized operations, flexible logistics, and proactive donor engagement ensured continuity of DHM services in both crises. Their findings offer a practical model for integrating milk banking into emergency preparedness frameworks, which is particularly crucial for the most vulnerable infants.

## Conclusion: a call to action

This editorial and the contributions to this Research Topic reaffirm a critical truth: when maternal breastfeeding is not possible, the human right of every infant to receive safe, nutritious, and appropriate feeding must not be compromised. Human milk, whether through maternal expression, wet nursing, or donor human milk, is not merely an alternative; it is a life-saving intervention that must be prioritized, safeguarded, and systematically supported (46).

Infant formula may be necessary in rare circumstances. In those situations, its use must be governed by strict protocols, and only in the context of skilled counseling on breastfeeding, relactation, wet nursing, and strong monitoring mechanisms to prevent harm (7). Unregulated or inappropriate formula use, especially in emergency settings, may seem an immediate and convenient solution but results in serious health risks and long-term consequences for infant survival and development (35–38).

In an era of escalating humanitarian emergencies, pandemics, and climate-induced displacement, infant and young child feeding (IYCF) is a lifesaving intervention. It cannot be an afterthought. Global and national actors must urgently operationalize the *OG-IFE* as a core element of emergency preparedness, health system resilience, and humanitarian response.

Central to this agenda is the urgent need to invest consistently and on a scale in activities that promote, protect, and support breastfeeding, and ensure access to safe, age-appropriate complementary feeding while continuing breastfeeding for at least 2 years. This includes building the capacity of frontline workers to support breastfeeding, relactation and wet nursing, establishing human milk banking systems if necessary, supporting community-based counseling networks, and ensuring families have the information, resources, and environments they need to nourish their children.

The evidence, innovations, and experiences presented in this series offer a clear, actionable roadmap. The global community must respond with political will, financial commitment, and coordinated action. The time for debate has passed. We must act decisively and collectively—to uphold the nutritional rights of every infant and young child, in every context.
